# Impact of Passive Smoking on Vital Signs, Motor Activity, and Agitation in Children Undergoing Dental Extractions Under Sedation: A Short-Term Cohort Study

**DOI:** 10.3390/healthcare14111451

**Published:** 2026-05-24

**Authors:** Elif Buse Kaplan, Aysun Avşar

**Affiliations:** Department of Pediatric Dentistry, Faculty of Dentistry, Ondokuz Mayıs University, 55280 Samsun, Türkiye; aysuna@omu.edu.tr

**Keywords:** passive smoking, pediatric dentistry, dental sedation, perioperative monitoring, oxygen saturation, emergence delirium, recovery time

## Abstract

Background and Aim: Passive smoking (PS) is a well-established risk factor associated with systemic and oral health impairments in children. However, its influence on perioperative physiological stability and recovery profiles during pediatric dental sedation remains insufficiently elucidated. This study investigated the association between PS exposure and perioperative vital parameters, recovery characteristics, and emergence behavioral outcomes in children undergoing dental extractions under sedation. Methods: This prospective cohort study (ClinicalTrials.gov: NCT06780189) included 100 ASA I children aged 4–6 years scheduled for primary molar extraction under midazolam-remifentanil-propofol sedation. Participants were stratified into three groups: no exposure, caregiver and household exposure, and household exposure only. An exposure-related relationship was evaluated based on daily household cigarette consumption. Perioperative vital signs (HR, blood pressure, and SpO_2_) were continuously monitored. Postoperative recovery and emergence profiles were assessed using the Modified Aldrete Recovery Score (MASS), Richmond Agitation–Sedation Scale (RASS), and Pediatric Anesthesia Emergence Delirium (PAED) scale. Results: Children exposed to PS demonstrated significantly lower SpO_2_ levels across all perioperative phases compared with non-exposed counterparts (*p* < 0.001), reflecting an exposure-related effect. In contrast, no statistically significant differences were observed in cardiovascular parameters (*p* > 0.05). Recovery time was significantly prolonged in PS-exposed children (*p* = 0.002). Furthermore, PS exposure was associated with significantly higher RASS and PAED scores, indicating increased agitation and emergence delirium (*p* < 0.001). Conclusions: Passive smoking adversely affects perioperative oxygenation, delays recovery, and exacerbates emergence neurobehavioral disturbances in children undergoing dental sedation. Environmental tobacco exposure must be integrated into preoperative risk assessments.

## 1. Introduction

Tobacco use remains one of the leading global public health challenges, accounting for more than eight million deaths annually, including approximately 1.6 million deaths among non-smokers exposed to second-hand smoke [1]. Despite heightened awareness of its detrimental health effects and the introduction of increasingly restrictive tobacco control policies in many countries, consumption persists at alarming levels. In 2019, there were an estimated 1.3 billion tobacco users worldwide, with nearly 80% residing in low- and middle-income countries, where weaker health infrastructures and limited regulatory capacity render populations particularly vulnerable to the strategies of the tobacco industry including marketing practices, targeting of vulnerable populations, and misinformation [1,2]. This pervasive use not only endangers smokers themselves but also imposes substantial health risks on children through passive exposure, which remains among the most common and preventable environmental toxicants during early life [2].

Passive smoking can be classified into two subcategories: secondhand smoke, defined as the combination of sidestream smoke from the burning end of a cigarette and mainstream smoke exhaled by smokers, and thirdhand smoke, which refers to residual nicotine and other harmful chemicals adhering to indoor surfaces, dust, clothing, and furniture long after active smoking has ceased [3]. Both forms of exposure are of particular concern in pediatric populations, as children may be continuously subjected to toxic compounds even without direct contact with active smokers [3,4]. Passive smoking, also termed secondhand smoke (SHS) or environmental tobacco smoke (ETS), refers to the involuntary inhalation of tobacco combustion products by non-smokers, and is recognized as a major preventable risk factor for respiratory, cardiovascular, and neoplastic diseases [2,3].

In 2004, it was estimated that nearly 40% of children worldwide were exposed to passive smoking, and more recent epidemiological studies suggest that these rates remain alarmingly high. Recent surveys have shown that approximately one-quarter of middle and high school students reported exposure to passive smoking within their homes [5,6]. In Turkey, the situation appears even more concerning, as more than half of children aged 0–12 years are exposed to at least one smoker in the household, with the prevalence exceeding 70% in socioeconomically disadvantaged regions [7,8]. Although national bans have successfully reduced exposure in workplaces and public spaces, exposure within households remains largely unchanged, thus representing the primary source of risk for children [9].

Children between 4 and 6 years of age are particularly vulnerable owing to immature alveolar structures, narrower airway calibers, insufficient mucociliary clearance, and weaker immune defenses [10,11]. The adverse health consequences of passive smoking are multifaceted. In children, it has been consistently associated with impaired lung function, asthma, recurrent respiratory infections, and allergic conditions [12,13]. Furthermore, prolonged exposure has been linked to an increased risk of cardiovascular morbidity in later life, including hypertension, metabolic syndrome, myocardial infarction, and stroke [14]. Mechanistic studies have demonstrated that oxidative stress, endothelial dysfunction, and platelet activation are central to these processes. Notably, the activation of nicotinamide adenine dinucleotide phosphate oxidase-2 (NOX-2) has been identified as a key driver in the pathogenesis of atherothrombotic disease, and passive smoking has been shown to activate similar pathogenic pathways in children, thereby predisposing them to premature atherosclerosis [15,16].

Beyond systemic outcomes, the oral health consequences of passive smoking warrant particular attention. A growing body of evidence indicates that children exposed to tobacco smoke experience a higher prevalence of dental caries. This phenomenon has been attributed to reductions in salivary pH, suppression of salivary immunoglobulins such as IgA, disturbances in the oral microbial balance, and diminished remineralization capacity [17,18]. In addition, passive smoking in children has been significantly associated with the development of gingival pigmentation and long-term exposure has been associated with structural and developmental dental abnormalities, including delayed dental age, enamel hypoplasia, and developmental dental malformation These findings underscore that passive smoking exerts a profound impact not only on systemic health but also on oral development, thereby compromising overall well-being during critical stages of growth [17].

In pediatric dentistry, sedation represents an indispensable modality, particularly for children with high levels of anxiety or limited cooperation [19]. Recovery from sedation—including emergence and the time to regain consciousness—can be prolonged or disrupted in children with systemic conditions such as obesity, asthma, or attention-deficit/hyperactivity disorder (ADHD), which influence pharmacokinetics, airway reactivity, and perioperative behavior [20,21]. These alterations can manifest as irregularities in vital parameters, prolonged recovery times, and an increased incidence of perioperative complications. However, despite the well-documented systemic and oral consequences of passive smoking, the literature remains markedly limited in addressing its potential influence on pediatric sedation safety and clinical outcomes.

Consequently, our study seeks to bridge this critical gap in the literature by systematically investigating the effects of passive smoking exposure on sedation processes in children. By doing so, we aim not only to provide novel insights into the biological and clinical implications of passive smoking but also to inform and improve pediatric dental care and sedation safety in this particularly vulnerable population.

## 2. Materials and Methods

### 2.1. Study Population and Sample

This clinical study was conducted between April 2024 and July 2024 at the Department of Pediatric Dentistry and the Department of General Anesthesia, Faculty of Dentistry, Ondokuz Mayıs University. The study population consisted of children aged 4–6 years who demonstrated non-cooperative behavior, presented with an indication for primary tooth extraction, and were scheduled for treatment under general anesthesia.

The sample size calculation was performed using G*Power version 3.1.9.4 (Heinrich-Heine-Universität Düsseldorf, Düsseldorf, Germany; licensed to Ondokuz Mayıs University) with reference to the study by Torun et al. [22], utilizing heart rate (HR) as the primary outcome variable due to its clinical variance in sedation profiles. For a One-way Analysis of Variance (ANOVA) across three independent exposure groups, assuming a medium-to-large effect size (Cohen’s f = 0.375), a significance level (alpha) of 0.05, and a high statistical power (1 − beta) of 0.95, the minimum required sample size was determined to be 96 participants. To account for potential data dropouts or attrition, a total of 100 patients (*n* = 100) were enrolled, ensuring that the study maintains a robust statistical power well above the 95% threshold to detect clinically meaningful differences.

Prior to enrollment, all children and their parents were informed about the study procedures. Written informed consent was obtained from parents or legal guardians who voluntarily agreed to participate. In addition to written parental informed consent, verbal informed assent was obtained from all capable pediatric participants after explaining the procedures in an age-appropriate manner, ensuring strict compliance with ethical standards for pediatric research.

### 2.2. Ethical Approval and Clinical Trial Registration

The study protocol was approved by the Ondokuz Mayıs University Clinical Research Ethics Committee (Protocol No: 2023/387, approval date: 22 November 2023). The trial was registered at ClinicalTrials.gov (Identifier: NCT06780189). All procedures were conducted in accordance with the ethical standards of the institutional research committee and the principles of the 1964 Helsinki Declaration and its later amendments. The reporting of this study conforms to the Strengthening the Reporting of Observational Studies in Epidemiology (STROBE) guidelines for cohort studies [23].

### 2.3. Inclusion and Exclusion Criteria

Children aged 4–6 years, classified as ASA I according to the American Society of Anesthesiologists (ASA) classification, and exhibiting scores of 1 or 2 on the Frankl Behavior Rating Scale were included. Eligible participants were required to present with an indication for the extraction of at least two primary maxillary molars and to provide a signed informed consent form.

Children with systemic health conditions classified as ASA II or higher, those with Frankl scores of 3 or 4, and children outside the defined age range were excluded. Additional exclusion criteria comprised the presence of acute apical abscesses or cystic lesions, indications for permanent tooth extraction, parental use of electronic cigarettes or nicotine replacement therapy, and refusal to participate.

### 2.4. Rationale for Inclusion and Exclusion Criteria

The age range of 4–6 years was selected because children in this developmental stage frequently demonstrate limited cooperation during dental treatment and are particularly vulnerable to the systemic and behavioral effects of passive smoking. Restricting the study population to ASA I patients minimized potential confounding factors related to systemic health that could influence sedation outcomes. The inclusion of children with Frankl scores of 1 or 2 ensured the selection of patients with definitely negative or negative behavior, who represent the primary candidates for sedation or general anesthesia in pediatric dentistry [19]. Requiring at least two primary maxillary molar extractions provided procedural standardization, as these interventions typically necessitate comparable treatment duration and sedation depth. Exclusion criteria, such as systemic comorbidities, acute odontogenic infections, or permanent tooth extraction, were applied to avoid additional variables that might compromise the reliability of the results.

### 2.5. Data Collection Process

Children’s exposure to passive smoking was assessed through a six-item parent-administered questionnaire, adapted from instruments originally developed by Groner et al. and Avşar et al. [18,24]. The overall reliability coefficient (Cronbach’s Alpha) for the scale was found to be 0.923, and the reliability coefficients for each sub-dimension were above 0.80 which indicated excellent fit [25].

The questionnaire addressed the duration of the child’s stay at home, the identity of the primary caregiver, the smoking habits of caregivers and other household members, and the total number of cigarettes consumed at home (≤10, 10–20, ≥20). Based on the responses, participants were categorized into three groups: Group 1, neither the caregiver nor household members smoked; Group 2, both the caregiver and household members smoked; and Group 3, only household members smoked while the caregiver did not.

### 2.6. Sedation Protocol and Monitoring Protocol

Upon admission to the operating room, each child was connected to standard monitoring devices, and heart rate (HR), systolic arterial pressure (SAP), diastolic arterial pressure (DAP), mean arterial pressure (MAP), and peripheral oxygen saturation (SpO_2_) were recorded. Measurements were obtained before induction of anesthesia, during the administration of local anesthesia, and at 5 min intervals throughout the procedure. Intraoperative complications, such as airway obstruction, were also documented.

An intravenous line was established using a 22G (Beybi, Istanbul, Turkey)cannula, and infusion with 3.33% dextrose containing 0.3% NaCl solution was initiated. Continuous monitoring of electrocardiography, blood pressure, and SpO_2_ was maintained throughout the procedure. Anesthesia induction was performed with midazolam (0.03 mg/kg IV), remifentanil (0.5 µg/kg IV), and propofol (1 mg/kg IV) (Sandoz, Germany) [26]. For children presenting with a Richmond Agitation–Sedation Scale (RASS) score ≥2, propofol was repeated as a maintenance dose at 0.5 mg/kg IV. The RASS was also employed continuously to standardize the assessment of sedation depth and agitation, with scores ranging from +4 (combative) to −5 (unarousable), where 0 represented an alert and calm state [27].

Once patients were placed under deep sedation, infiltrative local anesthesia was administered, followed by the extraction of the indicated primary teeth. Local anesthesia was achieved using a plain 3% mepivacaine (Safodont, Istanbul, Turkey) solution (without epinephrine) to eliminate potential confounding effects of exogenous vasoconstrictors on the recorded cardiovascular parameters, thereby allowing a more direct assessment of the relationship between passive smoking and perioperative vital signs. After each of these procedural steps—induction, local anesthesia, and extraction—vital parameters, including HR, SAP, DAP, MAP, and SpO_2_, were systematically measured and recorded to ensure accurate documentation of hemodynamic and respiratory changes throughout the operative process [26].

### 2.7. Recovery and Agitation Assessment

Postoperative recovery was evaluated in the recovery unit. The Modified Aldrete Recovery Score (MASS) was applied at 5 min intervals, assessing activity, respiration, circulation, consciousness, and oxygen saturation. Each domain was scored between 0 and 2, yielding a maximum of 10 points. A total score of ≥9 was considered sufficient for safe discharge from the recovery area [28].

Agitation during recovery was assessed with the RASS, which provided a structured evaluation of both sedation depth and agitation using the same scoring system applied intraoperatively. In addition, the Pediatric Anesthesia Emergence Delirium (PAED) scale was used to quantify emergence delirium. This tool consists of five behavioral parameters: eye contact, purposeful actions, awareness of surroundings, restlessness, and inconsolability. Each item is scored from 0 to 4, resulting in a total score ranging from 0 to 20, with higher values indicating more severe delirium. The PAED scale was administered by trained observers at 5, 10, 15, and 20 min after transfer to the recovery room under standardized conditions with controlled noise and lighting [29].

The surgical team and the observers recording the PAED, MASS, and RASS scores were blinded to the smoking status and group allocations of the children to eliminate assessment bias.

### 2.8. Statistical Analysis

All statistical analyses were performed using SPSS software version 20.0 (licensed to Ondokuz Mayıs University). Normality of continuous variables was assessed using the Kolmogorov–Smirnov test, and homogeneity of variance was evaluated with Levene’s test. For parametric data, one-way ANOVA was applied, followed by Tukey–Kramer post hoc test for multiple comparisons. For categorical variables, the Kruskal–Wallis H test and Mann–Whitney U test were employed. Associations with additional drug administration were analyzed using the Chi-square test. Changes in vital parameters over time were examined using repeated-measures ANOVA.

## 3. Results

### 3.1. Demographic Characteristics

The study was completed with a total of 100 pediatric patients. The study population consisted of 51 males (51%) and 49 females (49%), with a mean age of 5.01 ± 0.75 years. Statistical analysis showed no significant differences between the groups regarding age or gender distribution (*p* > 0.05).

### 3.2. Preoperative and Intraoperative Vital Signs

As shown in Table 1, SpO_2_ values differed significantly across exposure groups; in contrast no significant intergroup differences were observed in HR, SAP, DAP, or MAP at any perioperative time point (*p* > 0.05). Preoperatively, children from non-smoking households exhibited higher SpO_2_ levels (99.72 ± 0.08a) compared with those from households where 10–20 or ≥20 cigarettes were consumed daily (98.38 ± 0.24b and 98.43 ± 0.19b, respectively; *p* < 0.001). Similar differences persisted at IV bolus, after local anesthesia, and at both the 5th and 10th minutes intraoperatively (*p* < 0.05). These findings demonstrate that SpO_2_ decreased progressively with increasing total household cigarette consumption, reflecting an exposure-related effect. Regarding intraoperative safety, no complications such as airway obstruction, laryngospasm, or persistent desaturation were observed in any of the study participants across all groups.

As presented in Table 2, SpO_2_ values were consistently higher in Group 1 (neither caregiver nor household members smoked; 99.72 ± 0.08a) than in Group 2 (both caregiver and household members smoked; 98.27 ± 0.19b) and Group 3 (only household members smoked; 98.56 ± 0.15b) (all *p* < 0.001). This indicates that caregiver smoking, when combined with household exposure, exerts an additive detrimental effect on oxygenation.

Within caregiver consumption categories (Table 3), SpO_2_ values were highest among children whose caregiver did not smoke (99.13 ± 0.11a) and lowest among those exposed to caregivers smoking >20 cigarettes/day (98.17 ± 0.48b; *p* = 0.003). Intermediate strata (<10 or 10–20 cigarettes/day) were labeled “ab,” signifying no significant differences from either extreme. Significant differences were again observed intraoperatively at IV bolus (*p* = 0.026), after local anesthesia (*p* < 0.001), and at 5 min (*p* = 0.021), while no difference was detected at 10 min (*p* = 0.624).

As shown in Table 4, smoking by other household members also reduced SpO_2_ values. Children from non-smoking households maintained significantly higher oxygenation (99.68 ± 0.09a) than those exposed to <10, 10–20, or >20 cigarettes/day (all ≈98.3b; *p* < 0.001), whereas HR, SAP, DAP, and MAP remained unaffected (all *p* > 0.05).

Across all models, non-exposed children maintained significantly higher oxygenation, while greater exposure was associated with progressively lower SpO_2_ throughout the procedure.

### 3.3. Postoperative Agitation (RASS)

As shown in Table 5, RASS scores did not differ significantly at the 5th, 10th, or 15th minutes of recovery, with most children remaining in deep sedation (median −4, *p* > 0.05). However, at the 20th minute, non-exposed children had a median score of 0, while those exposed to household smoking exhibited persistent agitation (median 1; *p* < 0.001).

### 3.4. Postoperative Recovery (MASS)

As presented in Table 6, recovery time, defined as the interval to achieve a MASS ≥9, differed significantly across groups. Children from non-smoking households (1. Group) recovered more rapidly (median 15.5 min, range 10–20b) than those whose caregivers smoked (3. Group) (17 min, range 13–23a; *p* = 0.002). Similar patterns were observed when stratifying by other household members’ consumption (*p* = 0.005) and by total household cigarette use (*p* = 0.006). In contrast, no significant differences were detected when only caregiver cigarette counts were considered (*p* = 0.448).

### 3.5. Emergence Delirium (PAED)

PAED scores varied significantly across groups, as presented in Table 6. At the 5th minute, non-exposed children demonstrated markedly lower scores (mean 4–6b) compared with those from smoking households (mean 12–15a; *p* < 0.001). This difference persisted at the 10th, 15th, and 20th minutes, although scores gradually decreased in all groups. By the 20th minute, PAED scores approached baseline in the non-exposed group (3.0 ± 0.5b) but remained elevated among exposed children (9–10a; *p* < 0.001). Stratification by caregiver consumption, other household members, and total household use revealed consistent dose–response patterns, with greater exposure yielding more severe and persistent delirium.

As demonstrated in Table 6, combined evaluation of MASS, RASS, and PAED confirmed that passive smoking exposure was associated with delayed recovery, heightened agitation, and increased emergence delirium. Non-exposed children consistently exhibited shorter recovery times, lower PAED scores, and calmer RASS scores at 20 min, whereas exposed children experienced prolonged recovery, persistently elevated PAED values, and agitation during emergence.

## 4. Discussion

In this study, intraoperative vital parameters (SpO_2_, MAP, SBP, DBP, HR) and postoperative recovery indices (RASS, PAED, MASS) were evaluated in pediatric patients undergoing dental extraction under sedation who were exposed to passive smoking. A survey of the existing literature revealed to the best of our knowledge, limited data exist on research directly addressing the physiological and behavioral effects of passive smoke exposure in the context of pediatric dental sedation, underscoring the novelty and contribution of the present investigation.

Sedation remains indispensable in pediatric dentistry, particularly for children who present with poor cooperation or heightened anxiety. According to the 2019 AAPD guidelines, the successful and safe delivery of painful dental interventions often requires not only anxiolysis but also adequate immobility [26]. Minimal sedation protocols, however, may be insufficient for very young children or those with neurodevelopmental disorders. In such cases, intravenous sedation offers a more predictable and controllable option [30]. In the present study, propofol was selected due to its rapid onset, short half-life, and antiemetic properties. Nonetheless, despite its favorable pharmacological profile, propofol retains the potential for serious cardiopulmonary adverse effects, highlighting the necessity of administration by skilled anesthesiologists [30,31].

Children are particularly vulnerable to the pulmonary consequences of passive smoking owing to their higher respiratory rates, narrower airways, and mucosal hyper-reactivity. Carbon monoxide and nicotine impair hemoglobin’s oxygen-carrying capacity, thereby precipitating hypoxemia and compounding the risk of sedation-induced respiratory depression [32]. In this study, children exposed to passive smoking demonstrated significantly lower SpO_2_ levels, a finding consistent with Jaakkola and Gissler, who reported that passive smoke reduces peripheral oxygenation and undermines pulmonary function [33]. A numerical difference in SpO_2_ levels (e.g., 99.72% vs. 98.27%) may appear clinically subtle in a conscious individual; however, it carries profound implications under deep sedation where pharmacological respiratory depression is present. This statistically significant difference (*p* < 0.001) reflects a diminished oxygen reserve in children exposed to passive smoking. Within this clinical context, a lower baseline saturation narrows the safety margin, indicating that these patients are at a substantially higher risk for rapid desaturation to critical hypoxic levels in the event of an airway obstruction or hypoventilation. Physiologically, this suggests that the chronic inhalation of carbon monoxide and nicotine has already compromised the hemoglobin’s oxygen-carrying capacity. Therefore, these minor numerical deviations should be interpreted as a vital clinical warning sign of heightened pulmonary fragility during pediatric sedation. Moreover, MASSs indicated that children with lower oxygen saturation required significantly longer recovery times, suggesting that the deleterious effects of passive smoking extend beyond the intraoperative phase to impede postoperative recovery.

The 4–6-year age group emerged as particularly vulnerable to the respiratory consequences of passive smoking. At this developmental stage, the airways are still immature, rendering disruptions in oxygen transport more readily translated into clinically significant hypoxemia. These results highlight the heightened pulmonary fragility of younger children. In line with previous reports that passive smoking induces vasoconstriction and inflammatory cascades, our findings suggest that these pathophysiological mechanisms exert greater clinical impact during early childhood. Thus, in children aged 4–6 years, sedation planning should involve proactive anticipation of supplemental oxygen requirements and vigilant monitoring for respiratory depression.

In contrast, no statistically significant differences were observed in systolic or diastolic blood pressure, mean arterial pressure, or heart rate between groups. This suggests that the acute impact of passive smoking manifests predominantly in respiratory rather than cardiovascular physiology. Nonetheless, Aryanpur et al. identified passive smoking as a risk factor for pediatric hypertension, implying that long-term cardiovascular monitoring of exposed children remains warranted [34].

The findings also revealed that children exposed to passive smoking exhibited higher postoperative RASS and PAED scores, indicating impaired neurological emergence characterized by agitation and an increased risk of delirium. In households with higher tobacco consumption, PAED scores remained elevated during the early recovery phase, suggesting exacerbated behavioral instability. These observations are consistent with Chiswell et al., who linked passive smoke exposure to anesthesia-related complications, including laryngospasm and agitation [35]. MASSs in our study further corroborated these findings, showing that exposed children experienced significantly delayed recovery, reflecting adverse effects on neurophysiological processes. Importantly, an exposure-related relationship was evident, whereby the severity of behavioral and neurological disturbances increased with greater smoke exposure. In the present study, although SpO_2_ levels were significantly lower in passive smokers compared to non-smokers, a strictly linear dose–response relationship regarding the number of cigarettes was not statistically confirmed. This lack of significance between different consumption strata could be attributed to the relatively limited sample size in sub-categories, which may have reduced the statistical power to detect subtle gradients. Furthermore, it is plausible that caregivers may have underreported the actual number of cigarettes consumed due to social desirability bias, as they are often aware of the detrimental effects of tobacco smoke on child health. Consequently, the physiological impact observed in this cohort appears to function as an exposure-related threshold effect rather than a clearly defined dose-dependent correlation.

The 4–6-year age group warrants special consideration in this regard. At this stage of development, the central nervous system remains in maturation, producing variable and less predictable pharmacodynamic responses to sedative agents [36]. Our results demonstrated that children within this age group exhibited significantly higher RASS and PAED scores, supporting the hypothesis that nicotine and carbon monoxide disrupt GABAergic neurotransmission, thereby aggravating agitation and behavioral dysregulation [37]. In particular, children whose primary caregivers smoked showed persistently elevated PAED scores, which translated into an increased risk of postoperative delirium. Moreover, MASSs reached the recovery threshold “9” more slowly in these children, indicating that neurophysiological recovery was substantially delayed. Collectively, these findings suggest that children aged 4–6 years exhibit significant susceptibility to both respiratory and neurological complications arising from passive smoke exposure, warranting vigilant monitoring during the perioperative period.

Postoperative anxiety scores were significantly higher in smoke-exposed children, plausibly explained by the deleterious effects of nicotine and other toxicants on GABA neurotransmission. This finding accords with Bandiera et al., who reported increased risks of behavioral inhibition and anxiety disorders in children exposed to passive smoke [38]. Consequently, passive smoking appears to compromise not only physiological function but also psychological well-being in pediatric populations.

An additional observation of this study is that the adverse effects of passive smoking were not confined to parental smoking; rather, smoking by any household member exerted comparable effects on the child [39]. While maternal smoking often shows a stronger association with respiratory morbidity, cumulative exposure from multiple household smokers is likewise detrimental, amplifying risks across asthma, wheeze, and bronchitic symptoms [40]. Furthermore, the growing concern of third-hand smoke exposure—resulting from toxic residues persisting on surfaces such as furniture, toys, and walls—constitutes an additional hazard, particularly for preschool-aged children [41]. Such residual exposure may perpetuate respiratory and neurobehavioral risks long after active exposure has ceased.

The present findings highlight the detrimental impact of passive smoking on children undergoing dental procedures under sedation. Nevertheless, several limitations should be acknowledged. Firstly, the study was conducted at a single center with a relatively small sample size (*n* = 100), which limits the generalizability of the results. Secondly, passive smoking exposure was determined solely through parental self-reports rather than objective biochemical verification (e.g., measuring cotinine levels in saliva or urine). This reliance on self-reported data may have introduced social desirability bias, leading to an imprecise categorization of exposure doses and potentially obscuring a clearer dose–response relationship. Future studies incorporating biochemical validation and larger cohorts are necessary to confirm potential dose-dependent physiological responses more accurately.

Thirdly, a significant technical constraint is that standard pulse oximetry cannot distinguish oxyhemoglobin from carboxyhemoglobin. In children exposed to passive smoke, elevated carboxyhemoglobin levels may lead to an overestimation of actual arterial oxygen saturation. The absence of co-oximetry or exhaled carbon monoxide measurement remains a constraint for a more precise characterization of oxygenation in this population. Furthermore, our analysis did not incorporate a multivariate model to account for potential confounding variables such as procedure duration, body weight, and exact age within the 4–6-year range.

Fourthly, the investigation focused only on short-term outcomes, without evaluating potential long-term respiratory or neuropsychological effects. Another limitation of our study is the absence of a standalone group representing only caregiver exposure without other household smokers. Due to the demographic characteristics of our cohort, primary caregiver smoking was almost invariably accompanied by smoke exposure from other household members. Consequently, to maintain adequate group sizes and ensure robust statistical power, these cases were integrated into the combined exposure group to maintain statistical power. Future studies with larger, more diverse populations could isolate this specific subgroup to more precisely delineate the independent impact of primary caregiver smoking. Finally, the number and location of dental extractions were not standardized, potentially contributing to variability in procedure duration and clinical responses. Despite these limitations, the study provides meaningful preliminary evidence and underscores the need for larger, multi-center studies with standardized methods and objective exposure assessments.

## 5. Conclusions

In conclusion, the present study demonstrates that passive smoke exposure in children undergoing sedation for dental treatment adversely affects not only respiratory function but also neurological and behavioral recovery. The observed exposure-related associations further reinforce the clinical importance of considering environmental tobacco exposure during perioperative risk assessment. Accordingly, beyond age, weight, and medical history, passive smoke exposure should be incorporated as a critical determinant in sedation planning. Such an approach is essential for mitigating perioperative complications and ensuring a safer postoperative recovery trajectory.

## Figures and Tables

**Table 1:** Vital signs according to the number of cigarettes smoked daily in the household.

Number of Cigarettes Smoked Daily in the Household	*n*	HR	SAP	DAP	MAP	SpO_2_
<10	21	106.29 ± 3.68	114.76 ± 3.05	70.14 ± 2.15	85.67 ± 2.32	98.43 ± 0.20 ^b^
10–20	13	113.08 ± 5.96	114.54 ± 4.49	65.00 ± 2.84	81.85 ± 2.76	98.38 ± 0.24 ^b^
>20	30	105.90 ± 3.22	115.73 ± 2.75	67.00 ± 2.41	86.77 ± 2.62	98.43 ± 0.19 ^b^
None	36	111.67 ± 2.98	119.25 ± 1.90	72.36 ± 1.79	89.36 ± 1.85	99.72 ± 0.08 ^a^
*p*		0.429	0.552	0.125	0.200	<0.001
	Intraoperative IV Bolus Vital Parameters					
<10	21	94.48 ± 3.90	104.43 ± 3.04	57.29 ± 2.32	72.86 ± 2.39	98.38 ± 0.28 ^b^
10–20	13	100.23 ± 4.29	99.54 ± 3.60	56.23 ± 2.58	72.15 ± 2.78	98.00 ± 0.20 ^b^
>20	30	101.57 ± 3.51	102.13 ± 2.13	56.00 ± 1.68	72.00 ± 1.87	98.13 ± 0.23 ^b^
None	36	100.03 ± 3.18	98.58 ± 1.77	55.69 ± 1.23	68.86 ± 1.55	99.78 ± 0.07 ^a^
*p*		0.580	0.309	0.932	0.425	<0.001
	Intraoperative Vital Parameters Following Local Anesthesia Administration					
<10	21	94.10 ± 2.79	96.48 ± 2.41	54.90 ± 1.52	68.76 ± 1.65	98.38 ± 0.23 ^b^
10–20	13	102.69 ± 4.39	95.77 ± 3.15	53.62 ± 1.68	67.85 ± 2.05	97.85 ± 0.19 ^b^
>20	30	99.57 ± 2.36	101.83 ± 2.39	57.60 ± 2.10	74.33 ± 2.26	97.73 ± 0.19 ^b^
None	36	101.42 ± 2.47	97.28 ± 1.75	54.89 ± 1.51	69.22 ± 1.63	99.39 ± 0.10 ^a^
*p*		0.221	0.244	0.504	0.095	<0.001
	Vital Parameters at the 5th Minute of the Intraoperative Procedure					
<10	21	114.86 ± 3.47	112.67 ± 2.64	65.67 ± 2.44	82.00 ± 2.79	98.23 ± 0.38 ^b^
10–20	13	113.92 ± 3.27	111.31 ± 4.11	62.92 ± 2.61	81.31 ± 3.37	97.73 ± 0.22 ^bc^
>20	30	113.17 ± 2.55	114.23 ± 2.09	64.77 ± 2.02	81.70 ± 2.12	97.33 ± 0.27 ^c^
None	36	112.42 ± 2.58	110.47 ± 2.39	62.22 ± 1.53	80.17 ± 2.02	99.33 ± 0.11 ^a^
*p*		0.942	0.700	0.591	0.939	<0.001
	Vital Parameters at the 10th Minute of the Intraoperative Procedure					
<10	21	110.25 ± 6.05	120.50 ± 10.60	73.75 ± 7.44	91.75 ± 7.47	98.00 ± 0.71 ^ab^
10–20	13	110.00 ± 4.00	110.50 ± 1.50	59.00 ± 3.00	77.50 ± 4.50	97.75 ± 1.00 ^ab^
>20	30	119.67 ± 3.71	122.67 ± 11.68	67.00 ± 9.07	84.33 ± 9.87	97.00 ± 0.25 ^b^
None	36	126.33 ± 5.84	109.33 ± 11.22	61.67 ± 9.87	80.33 ± 9.21	99.67 ± 0.33 ^a^
*p*		0.197	0.778	0.635	0.673	0.046

Values with different superscript letters (a, b, c) are significantly different among groups at *p* < 0.05.

**Table 2:** Vital signs according to the tobacco use among primary caregivers and other household members.

Tobacco Use Among Primary Caregivers and Other Household Members	*n*	HR	SAP	DAP	MAP	SpO_2_
	Preoperative Vital Parameters					
1. Group	36	111.67 ± 2.98	119.25 ± 1.90	72.36 ± 1.79	89.36 ± 1.85	99.72 ± 0.08 ^a^
2. Group	30	104.93 ± 3.40	114.20 ± 3.05	65.60 ± 2.34	84.50 ± 2.48	98.27 ± 0.19 ^b^
3. Group	34	109.74 ± 3.03	116.03 ± 2.22	69.61 ± 1.76	86.21 ± 1.94	98.56 ± 0.15 ^b^
*p*		0.311	0.317	0.052	0.247	<0.001
	Intraoperative IV Bolus Vital Parameters					
1. Group	36	100.03 ± 3.18	98.58 ± 1.77	55.69 ± 1.23	68.86 ± 1.55	99.78 ± 0.07 ^a^
2. Group	30	96.90 ± 3.36	102.23 ± 2.14	55.40 ± 1.81	71.73 ± 1.88	98.23 ± 0.24 ^b^
3. Group	34	100.79 ± 3.07	102.47 ± 2.31	57.41 ± 1.59	72.82 ± 1.78	98.15 ± 0.18 ^b^
*p*		0.677	0.324	0.609	0.235	<0.001
	Intraoperative Vital Parameters Following Local Anesthesia Administration					
1. Group	36	101.42 ± 2.47	97.28 ± 1.75	54.89 ± 1.51	69.22 ± 1.63	99.39 ± 0.10 ^a^
2. Group	30	99.70 ± 2.37	101.90 ± 2.43	57.77 ± 2.18	73.67 ± 2.33	97.73 ± 0.18 ^b^
3. Group	34	97.26 ± 2.47	96.15 ± 1.84	54.26 ± 0.98	69.00 ± 1.24	98.18 ± 0.18 ^b^
*p*		0.471	0.115	0.273	0.122	<0.001
	Vital Parameters at the 5th Minute of the Intraoperative Procedure					
1. Group	36	112.42 ± 2.58	110.47 ± 2.39	62.22 ± 1.53	80.17 ± 2.02	99.33 ± 0.11 ^a^
2. Group	30	112.17 ± 2.34	115.47 ± 2.21	65.60 ± 2.04	82.60 ± 2.38	97.73 ± 0.21 ^b^
3. Group	34	115.38 ± 2.59	111.06 ± 2.09	63.88 ± 1.77	80.94 ± 1.88	97.68 ± 0.24 ^b^
*p*		0.607	0.251	0.409	0.710	<0.001
	Vital Parameters at the 10th Minute of the Intraoperative Procedure					
1. Group	36	126.33 ± 5.84	109.33 ± 11.21	61.67 ± 8.97	80.33 ± 9.21	99.67 ± 0.33 ^a^
2. Group	30	114.00 ± 6.25	120.25 ± 8.61	68.25 ± 6.54	85.50 ± 7.08	98.00 ± 0.41 ^ab^
3. Group	34	112.80 ± 3.62	118.00 ± 8.56	68.20 ± 6.93	86.60 ± 6.98	97.00 ± 0.54 ^b^
*p*		0.213	0.734	0.813	0.848	0.015

Values with different superscript letters (a, b) are significantly different among groups at *p* < 0.05.

**Table 3:** Vital signs according to the number of cigarettes smoked daily by the primary caregiver.

Number of Cigarettes Smoked Daily by the Primary Caregiver	*n*	HR	SAP	DAP	MAP	SpO_2_
	Preoperative Vital Parameters					
<10	10	95.56 ± 4.47	113.11 ± 4.38	65.89 ± 1.90	85.11 ± 3.04	98.33 ± 0.41 ^ab^
10–20	14	107.21 ± 5.44	115.79 ± 5.20	65.57 ± 4.51	85.57 ± 4.45	98.36 ± 0.20 ^ab^
>20	6	108.00 ± 8.37	114.00 ± 6.65	67.83 ± 4.51	82.33 ± 5.60	98.17 ± 0.48 ^b^
None	70	111.13 ± 2.08	117.48 ± 1.47	70.63 ± 1.27	87.68 ± 1.34	99.13 ± 0.11 ^a^
*p*		0.104	0.773	0.346	0.682	0.003
	Intraoperative IV Bolus Vital Parameters					
<10	10	87.44 ± 2.48	96.89 ± 4.49	52.89 ± 3.40	66.89 ± 3.15	98.67 ± 0.47 ^ab^
10–20	14	102.21 ± 4.94	103.50 ± 3.93	54.57 ± 3.19	73.21 ± 3.48	98.14 ± 0.31 ^ab^
>20	6	99.50 ± 10.86	101.50 ± 1.59	58.83 ± 2.18	72.17 ± 2.27	97.83 ± 0.65 ^b^
None	70	100.28 ± 2.18	100.99 ± 1.40	56.70 ± 0.97	71.08 ± 1.16	98.97 ± 0.13 ^a^
*p*		0.232	0.651	0.479	0.524	0.026
	Intraoperative Vital Parameters Following Local Anesthesia Administration					
<10	10	96.78 ± 3.66	100.00 ± 5.12	57.00 ± 4.12	74.44 ± 4.32	97.89 ± 0.48 ^ab^
10–20	14	100.93 ± 3.42	103.00 ± 4.20	60.07 ± 3.72	75.29 ± 4.06	97.86 ± 0.14 ^ab^
>20	6	99.33 ± 7.84	97.50 ± 4.65	53.50 ± 2.97	68.00 ± 3.35	97.33 ± 0.56 ^b^
None	70	99.56 ± 1.72	97.20 ± 1.20	54.63 ± 0.89	69.24 ± 1.00	98.77 ± 0.12 ^a^
*p*		0.925	0.374	0.200	0.116	<0.001
	Vital Parameters at the 5th Minute of the Intraoperative Procedure					
<10	10	112.56 ± 3.23	118.56 ± 5.21	69.11 ± 3.89	87.89 ± 4.36	97.44 ± 0.41 ^b^
10–20	14	110.43 ± 3.98	113.57 ± 2.33	64.00 ± 3.08	81.57 ± 2.71	98.00 ± 0.30 ^ab^
>20	6	115.5 0 ± 5.06	113.17 ± 5.86	63.17 ± 3.74	75.67 ± 6.61	97.33 ± 0.49 ^b^
None	70	113.85 ± 1.81	111.00 ± 1.58	63.14 ± 1.16	80.69 ± 1.38	98.54 ± 0.16 ^a^
*p*		0.852	0.407	0.432	0.237	0.021
	Vital Parameters at the 10th Minute of the Intraoperative Procedure					
<10	10	115.00 ± 12.00	120.5 ± 25.5	69.50 ± 15.50	88.00 ± 16.00	97.00 ± 1.00
10–20	14	115.00 ± 0.00	112.00 ± 0.00	56.00 ± 0.00	73.00 ± 0.00	97.00 ± 0.00
>20	6	117.00 ± 0.00	110.00 ± 0.00	60.00 ± 0.00	76.00 ± 0.00	98.00 ± 0.00
None	70	117.13 ± 4.27	117.00 ± 5.88	68.00 ± 5.15	86.38 ± 4.99	98.38 ± 0.53
*p*		0.996	0.969	0.855	0.784	0.624

Values with different superscript letters (a, b) are significantly different among groups at *p* < 0.05.

**Table 4:** Vital signs according to the daily cigarette consumption of other household members.

Daily Cigarette Consumption of Other Household Members	*n*	HR	SAP	DAP	MAP	SpO_2_
	Preoperative Vital Parameters					
<10	22	110.50 ± 3.54	117.18 ± 2.97	71.55 ± 3.02	87.41 ± 2.12	98.45 ± 0.19 ^b^
10–20	24	106.42 ± 4.00	111.83 ± 3.24	63.00 ± 5.72	82.25 ± 2.62	98.42 ± 0.17 ^b^
>20	16	107.50 ± 4.56	118.75 ± 3.27	69.75 ± 2.51	88.63 ± 3.53	98.31 ± 0.30 ^b^
None	38	110.37 ± 2.97	118.47 ± 1.97	71.87 ± 3.73	88.63 ± 1.84	99.68 ± 0.09 ^a^
*p*		0.812	0.259	0.052	0.192	<0.001
	Intraoperative IV Bolus Vital Parameters					
<10	22	94.73 ± 3.72	104.64 ± 2.87	56.64 ± 2.01	72.86 ± 2.21	98.27 ± 0.27 ^b^
10–20	24	100.46 ± 3.18	99.71 ± 2.97	56.08 ± 2.32	71.67 ± 2.48	98.13 ± 0.19 ^b^
>20	16	104.00 ± 5.55	102.25 ± 1.76	56.19 ± 1.29	71.88 ± 1.77	98.06 ± 0.35 ^b^
None	38	99.37 ± 3.06	99.18 ± 1.77	56.00 ± 1.32	69.32 ± 1.55	99.74 ± 0.07 ^a^
*p*		0.480	0.348	0.995	0.568	<0.001
	Intraoperative Vital Parameters Following Local Anesthesia Administration					
<10	22	96.32 ± 2.96	97.00 ± 2.20	53.91 ± 0.96	67.59 ± 1.26	98.32 ± 0.22 ^b^
10–20	24	100.33 ± 2.81	98.88 ± 2.93	56.42 ± 2.28	71.92 ± 2.53	97.83 ± 0.18 ^b^
>20	16	98.63 ± 3.60	100.19 ± 2.66	56.69 ± 2.52	74.13 ± 2.72	97.69 ± 0.28 ^b^
None	38	101.16 ± 2.35	97.84 ± 1.80	55.45 ± 1.55	69.71 ± 1.63	99.32 ± 0.11 ^a^
*p*		0.623	0.850	0.765	0.212	<0.001
	Vital Parameters at the 5th Minute of the Intraoperative Procedure					
<10	22	115.73 ± 3.35	112.14 ± 2.14	63.41 ± 1.99	79.91 ± 2.13	97.41 ± 0.27 ^b^
10–20	24	111.04 ± 2.94	111.88 ± 2.54	63.38 ± 1.89	81.13 ± 2.17	98.00 ± 0.28 ^b^
>20	16	115.94 ± 2.97	114.88 ± 3.42	66.81 ± 3.13	83.06 ± 3.50	97.69 ± 0.31 ^b^
None	38	112.34 ± 2.45	111.24 ± 2.41	63.03 ± 1.67	81.11 ± 2.13	99.24 ± 0.13 ^a^
*p*		0.603	0.831	0.644	0.889	<0.001
	Vital Parameters at the 10th Minute of the Intraoperative Procedure					
<10	22	110.25 ± 6.05	120.50 ± 10.60	73.75 ± 7.44	91.75 ± 7.47	97.00 ± 0.71 ^b^
10–20	24	111.67 ± 2.85	111.00 ± 1.00	58.00 ± 2.00	76.00 ± 3.00	97.67 ± 0.67 ^ab^
>20	16	122.00 ± 5.00	128.00 ± 18.00	72.50 ± 12.50	90.00 ± 14.00	98.00 ± 0.00 ^ab^
None	38	126.33 ± 5.84	109.33 ± 11.22	61.67 ± 9.87	80.33 ± 9.21	99.67 ± 0.33 ^a^
*p*		0.182	0.660	0.458	0.489	0.044

Values with different superscript letters (a, b) are significantly different among groups at *p* < 0.05.

**Table 5:** Comparison of RASS scores among exposure categories.

Tobacco Use Among Primary Caregivers and Other Household Members	*n*	RASS 5 min	RASS 10 min	RASS 15 min	RASS 20 min
1. Group	36	−4 (−4:−4)	−4 (−4:−1)	−4 (−4:0)	0 (0:0) ^b^
2. Group	30	−4 (−4:−4)	−4 (−4:−4)	−4 (−4:−4)	1 (1:1) ^a^
3. Group	34	−4 (−4:−4)	−4 (−4:−4)	−4 (−4:−4)	1 (1:1) ^a^
*p*		1.000	0.411	0.411	<0.001
Number of Cigarettes Smoked Daily by the Primary Caregiver					
<10	10	−4 (−4:−4)	−4 (−4:−4)	−4 (−4:−4)	1 (1:1) ^a^
10–20	14	−4 (−4:−4)	−4 (−4:−4)	−4 (−4:−4)	1 (1:1) ^a^
>20	6	−4 (−4:−4)	−4 (−4:−4)	−4 (−4:−4)	1 (1:1) ^a^
None	70	−4 (−4:−4)	−4 (−4:−1)	−4 (−4:0)	0 (0:1) ^b^
*p*		1.000	0.934	0.934	<0.001
Number of Cigarettes Smoked Daily by Other Household Members					
<10	22	−4 (−4:−4)	−4 (−4:−4)	−4 (−4:−4)	1 (1:1) ^b^
10–20	24	−4 (−4:−4)	−4 (−4:−4)	−4 (−4:−4)	1 (1:1) ^b^
>20	16	−4 (−4:−4)	−4 (−4:−4)	−4 (−4:−4)	1 (1:1) ^b^
None	38	−4 (−4:−4)	−4 (−4:−1)	−4 (−4:0)	0 (0:0) ^a^
*p*		1.000	0.652	0.652	<0.001
Number of Cigarettes Smoked Daily in the Household					
<10	21	−4 (−4:−4)	−4 (−4:−4)	−4 (−4:−4)	1 (1:1) ^a^
10–20	13	−4 (−4:−4)	−4 (−4:−4)	−4 (−4:−4)	1 (1:1) ^a^
>20	30	−4 (−4:−4)	−4 (−4:−4)	−4 (−4:−4)	1 (1:1) ^a^
None	36	−4 (−4:−4)	−4 (−4:−1)	−4 (−4:0)	0 (0:0) ^b^
*p*		1.000	0.620	0.620	<0.001

Superscript letters (a, b) indicate statistically significant differences in RASS scores among groups (*p* < 0.05).

**Table 6:** Comparison of MASS AND PAED scores among exposure categories.

Tobacco Use Among Primary Caregivers and Other Household Members	*n*	MASS Time	PAED 5 min	PAED 10 min	PAED 15 min	PAED 20 min
1. Group	36	15.5(10:20) ^b^	4 (0:8) ^b^	4 (0:6) ^b^	3.5 (1:5) ^b^	3 (0:5) ^b^
2. Group	30	15.5(15:19) ^a^	12(10:17) ^a^	11.5(10:15) ^a^	11 (10:15) ^a^	9 (9:13) ^a^
3. Group	34	17(13:23) ^a^	12 (9:17) ^a^	11 (7:16) ^a^	10 (7:14) ^a^	10 (7:13) ^a^
*p*		0.002	<0.001	<0.001	<0.001	<0.001
Number of Cigarettes Smoked Daily by the Primary Caregiver						
<10	10	17 (10:20)	12.5 (11:17) ^a^	12 (10:15) ^a^	10 (9:15) ^a^	10 (7:13) ^a^
10–20	14	16 (14:22)	13 (11:18) ^a^	12 (11:16) ^a^	11 (10:15) ^a^	10.5 (9:15) ^a^
>20	6	18 (15:29)	12 (10:14) ^a^	11.5 (9:14) ^a^	10.5 (7:13) ^a^	10.5 (7:12) ^a^
None	70	15(10:25)	8.5 (0:17) ^b^	6.5 (0:16) ^b^	5.5 (0:15) ^b^	5.5 (0–15) ^b^
*p*		0.448	<0.001	<0.001	<0.001	<0.001
Number of Cigarettes Smoked Daily by Other Household Members						
<10	22	16.5 (13:23) ^a^	11.5 (9:14) ^b^	10 (7:13) ^b^	10 (7:13) ^b^	9 (7:11) ^b^
10–20	24	17 (15:19) ^a^	13 (11:17) ^ab^	12 (10:16) ^ab^	11 (10:14) ^ab^	9.5 (9:12) ^b^
>20	16	15 (15:19) ^a^	15 (10:17) ^a^	15 (11:15) ^a^	14 (12:15) ^a^	13(12:13) ^a^
None	38	15.5 (10:20) ^b^	4 (0:8) ^c^	4 (0:6) ^c^	3.5 (1:5) ^c^	3 (0:5) ^c^
*p*		0.005	<0.001	<0.001	<0.001	<0.001
Number of Cigarettes Smoked Daily in the Household						
<10	21	16 (13:21) ^a^	11 (9:14) ^b^	10 (7:13) ^b^	10 (7:12) ^b^	9 (7:11) ^b^
10–20	13	18 (15:18) ^a^	14 (11:17) ^ab^	12 (10:16) ^a^	13 (11:14) ^ab^	10 (10:12) ^ab^
>20	30	16 (15:23) ^a^	13 (10:17) ^a^	12 (11:15) ^a^	12 (10:15) ^a^	11 (9:13) ^a^
None	36	15.5 (10:20) ^b^	4 (0:8) ^c^	4 (0:6) ^c^	3.5 (1:5) ^c^	3 (0:5) ^c^
*p*		0.006	<0.001	<0.001	<0.001	<0.001

Different superscript letters represent significant differences in MASS and PAED scores between exposure categories according to the Kruskal–Wallis H test (*p* < 0.05).

## Data Availability

The data presented in this study are available on reasonable request from the corresponding author. The data are not publicly available due to privacy and ethical restrictions related to the protection of pediatric participants’ clinical information.
